# Prevention of Catheter-Related Infections and Complications: A Narrative Literature Review of Vascular Care and Maintenance

**DOI:** 10.1155/ijvm/1427129

**Published:** 2025-08-01

**Authors:** Nathan T. Gilmore, Terrence Metz

**Affiliations:** ^1^Department of Critical Care, Hoag Hospital, Newport Beach, California, USA; ^2^Department of Radiology, Division of Interventional Radiology, Corewell Health William Beaumont University Hospital, Royal Oak, Michigan, USA

## Abstract

**Objectives:** This review assessed the burden of catheter-related infections (CRI), existing gaps in catheter care, and prevention recommendations for catheter-related bloodstream infections (CRBSIs). The review further discusses how the emergence of coronavirus disease (COVID-19) influenced CRBSI rates and prevention strategies in the post-COVID-19 era.

**Methods:** A targeted literature search was conducted of Embase, Ovid MEDLINE, and EBM Reviews. Where applicable, supplemental hand searches were performed to identify evidence for gaps in the targeted search results. The authors reviewed each study and selected those for inclusion based on the population, intervention, comparison, outcomes, and study design (PICOS) criteria. Relevant studies were assessed for inclusion in the present review.

**Results:** Both “active” methods (scrubbing, flushing, and locking) and “passive” methods (disinfection caps) have consistently been shown to reduce CRBSI risk when assessed individually. These practices have markedly improved CRBSI rates over the past two decades, although there are ongoing gaps in catheter care and adherence to best practices. COVID-19 reversed the trend towards improving CRBSI rates, and persistent challenges for nurse staffing and training have resulted in a failure to return to pre-COVID-19 CRBSI rates in the current post-COVID-19 era. These challenges are further compounded by limited rigorous comparative evidence assessing the relative efficacy of individual CRBSI prevention methods.

**Conclusions:** Improving adherence to hub disinfection, along with catheter care and maintenance protocols, is essential for the prevention of CRIs. Further, innovative approaches for simplifying protocols and “forcing function” may increase compliance with CRBSI prevention strategies. In our practice, we routinely use disinfection caps in addition to standard scrubbing and flushing, alongside increased training and monitoring procedures. Additional studies are needed to assess which individual or combination prevention strategies are most efficacious and feasible in the post-COVID-19 era.

## 1. Introduction

Vascular catheter insertion is the most frequent procedure among hospitalized patients [1]. However, all vascular catheters are associated with a risk of infection, given their tendency to become contaminated during use [1]. Disinfection of the catheter hub prior to line use and adherence to proper maintenance practices are essential for reducing bacterial colonization and preventing catheter-related infections [2].

Needleless connectors (NCs) provide an easily accessible entry point for flushing, aspiration, and infusion of fluids and/or medications, and they facilitate closure of a catheter line when not in use [3]. However, NCs are considered the cause of 50% of postinsertion CRIs and are used on almost all intravascular devices, including peripheral intravenous catheters (PIVCs), central venous catheters (CVCs), peripherally inserted central catheters (PICCs), and totally implantable vascular access devices (TIVADs) [2] when accessed. They are therefore a major focus for the prevention of CRIs and associated complications [2].

There are several products and methods currently used to reduce the risk of bacterial contamination of catheter hubs and NCs [2, 4–10]; however, there is uncertainty regarding which are most efficacious. Variations in recommendations for catheter maintenance, as well as suboptimal compliance with existing recommendations, have led to gaps in catheter care [2, 11, 12].

The objective of this narrative review is to summarize the available evidence on prevention methods and strategies used to reduce bacterial contamination of catheter hubs and NCs, and by extension, the risk of CRIs and their associated burden. Commonly recommended interventions are discussed, from passive disinfection methods, such as the use of disinfectant caps, to active methods, such as the “scrub the hub” protocol, to catheter flushing and locking. Current gaps in clinical care and challenges associated with adherence to prevention strategies are also considered in the context of reducing the risk of CRI and other complications. This review further explores how the emergence of coronavirus disease (COVID-19) influenced catheter-related bloodstream infection (CRBSI) rates and considers strategies for enhancing infection prevention in the post-COVID-19 era.

## 2. Materials and Methods

A targeted literature search was conducted on September 23, 2022, to capture published evidence related to the burden of central line–associated bloodstream infection (CLABSI), clinical and observational studies for prevention methods, and systematic reviews. Searches were conducted for the following databases: Embase (search period: 1974 to September 22, 2022), Ovid MEDLINE (1946 to September 22, 2022), and EBM Reviews (i.e., Cochrane Central Register of Controlled Trials [August 2022] and the Cochrane Database of Systematic Reviews [2005 to September 21, 2022]). Where applicable, supplemental hand searches were performed to identify evidence for gaps in the targeted search results (e.g., reviewing the bibliographies of relevant studies and subsequent publications that cited these studies). A total of 2563 records were identified across databases, including a total of 2481 unique records after deduplication. See the Supporting Information for the search strategy and results. A supplemental literature search was conducted in PubMed on October 14, 2022, and updated on April 29, 2025, to capture evidence for the COVID-19-related burden of CLABSI and associated prevention strategies (i.e., catheter-related infections [MeSH term] *or* CLABSI *or* CRBSI *and* COVID-19 [MeSH term; December 2019 to April 29, 2025]). The authors reviewed each study and selected those for inclusion based on the population, intervention, comparison, outcomes, and study design (PICOS) criteria. Relevant studies were assessed by both authors for inclusion in the present review. No formal quality or bias assessments were planned for included studies.

## 3. Results

### 3.1. Catheter-Related Infections and Complications

Vascular catheters are associated with a variety of delayed complications, including infection and device dysfunctions (e.g., CRI, occlusions, and phlebitis) [2, 13–15], which are the focus of this review. These complications often interrupt treatment for the underlying disease, increasing costs and placing further demands on hospital resources [13, 14]. Appropriate catheter care and maintenance, including flushing and locking, have been identified as a key preventative factor for complications [8, 15, 16].

Catheter-related infection, CRBSI, catheter-associated bloodstream infection (CABSI), and CLABSI are all terms used to describe intravascular catheter–related infections [17]. CLABSI is defined as a bloodstream infection not related to an infection at another site that develops within 48 h of a central line placement [18]. CRBSI is the preferred term used by the Infectious Diseases Society of America (IDSA) and requires a pathological diagnosis using positive blood cultures [17]. However, CRI, CRBSI, CABSI, and CLABSI are often used interchangeably; hereafter, these terms are presented in accordance with the studies cited.

Late-onset CLABSIs are typically associated with an intraluminal infection source and are often linked to suboptimal catheter care [19]. In contrast, early CLABSIs are typically associated with an extraluminal infection source and are consequently more commonly associated with insertion practices [19]. Most CLABSIs are late onset, indicating that poor catheter care is a key risk factor [20]. Given the connection between microbial load and infection [21, 22], the implementation of and strict adherence to disinfection of NCs, as well as catheter care and maintenance best practices, are essential to mitigating the risk of bacterial colonization of catheter hubs and CRIs [20].

There are two critical areas of practice to prevent microbial contamination via the catheter hub or NC: (1) use of aseptic technique and (2) effective catheter hub or NC disinfection prior to initial and subsequent line access. Several best practice strategies, such as dressings and sterile technique, are currently recommended to prevent the transmission of bacteria across hubs, thereby preventing intraluminal catheter colonization, biofilm formation, and bloodstream infection [1]. However, poor compliance with hub disinfection and flushing procedures is a significant risk factor for CLABSI, providing an opportunity for prevention where compliance can be improved, especially as CVCs are the most frequent source of bloodstream infections [23].

#### 3.1.1. Epidemiology of CLABSIs

In the United States, there are an estimated 30,000–80,000 preventable CLABSIs annually in intensive care units (ICUs) [24, 25], with a rate of 0.8 per 1000 central line days [26]. Globally, CLABSIs present a substantial risk for hospitalized patients, with pooled estimated mean occurrence rates of 4.4 CLABSIs per 100 inserted devices and 2.7 CLABSIs per 1000 catheter days [27].

During 2008–2016, CLABSI rates decreased by 44% in the United States following the introduction of several infection control measures [28]. This trend reversed during the early phase of the COVID-19 pandemic, with a 24% increase in overall CLABSI rates between 2019 and 2020 and with a 50% increase in ICUs [29].

#### 3.1.2. Clinical and Economic Burden of CLABSIs

CLABSIs are the most common hospital-acquired infections (HAIs) in ICUs [30] and lead to extended hospital stays, increased mortality risk, and additional healthcare costs [26]. With an estimated 7–13 days increase in ICU length of stay (LOS) and up to 25% increase in attributable mortality risk per infection [24, 31], CRBSIs impose a burden on healthcare facilities in terms of both bed occupancy and mortality, with CLABSIs responsible for the highest mortality rate of all HAIs [19, 32]. CRBSIs are associated with the highest cost burden of all HAIs, with the treatment cost thought to be up to $2.3 billion annually in the United States [24] and between £19.1 and £36.2 million annually in the United Kingdom [32]. Estimated treatment costs per CLABSI are presented in Table 1.

Despite the availability of effective preventative interventions [36], such as “scrub the hub,” compliance is inconsistent [2, 11, 12]. Several studies have conducted quality improvement initiatives focused on increasing compliance with catheter care recommendations, although relatively few have provided detailed descriptions of the added cost and staff time associated with these interventions. Among available studies reporting incremental costs and time, a 364-bed hospital implemented a formalized maintenance bundle observation process, termed *Rounds for Influence* (RfI), which recruited nurses to observe patients' nurses as they perform a maintenance bundle element (e.g., scrubs/dries for 15 s and disinfectant cap use). A decrease in the standardized infection ratio (SIR) was observed from 2017 to 2021 (0.9–0.53), accompanied by an increase in the number of RfI from approximately 50–350 [37]. Costs associated with quality improvement interventions are presented in Table 1.

### 3.2. Overview of CLABSI Prevention Strategies

Multiple products and methods are currently used to reduce the risk of bacterial contamination of catheter hubs or NCs and associated complications. Active disinfection methods include protocols such as “scrub the hub.” Passive disinfection methods include the use of disinfecting caps which are alcohol-impregnated covers placed upon access ports when not in use [4]. In addition, flushing and locking keep the catheter patent and reduce the risk of complications [4]. Often, a combination of these protocols is used (e.g., bundles). For example, the SASH (saline–administration–saline–heparin) protocol is widely used as the standard procedure for line access pre- and postmedication delivery, incorporating both flushing and locking. Individual prevention strategies are summarized below, including scrubbing, flushing, locking, disinfectant caps, and bundled approaches.

#### 3.2.1. Scrubbing-Based Prevention Strategies

The Center for Disease Control (CDC), the Society for Healthcare Epidemiology of America (SHEA), and the Infusion Nurses Society (INS) recommend that both catheter hubs and NCs be disinfected consistently and thoroughly before each intravenous device access (Table 2) [5–7]. “Scrub the hub”–based protocols, which involve manual scrubbing of the hub or NC with a disinfectant wipe, are the most common “active” method of hub disinfection [2]. While protocols vary in terms of friction applied, motion pattern used (rotational vs. back and forth), and air-drying time [2, 10, 39], all incorporate the use of an appropriate disinfectant such as 70% isopropyl alcohol (IPA), chlorhexidine gluconate (CHG), or povidone iodine [5, 6].

A systematic review of 10 studies examining NC decontamination found disinfectant wipes with 2% CHG and 70% IPA significantly reduced CLABSI risk relative to 70% IPA wipes alone (risk ratio: 0.28; 95% confidence interval [CI]: 0.20–0.39), although evidence was of low-to-mid quality [40]. A 2021 pilot study sought to generate feasibility data for comparing different types of disinfectant wipes for NC hub decontamination [41]. Low CLABSI rates were found for both 70% IPA wipes (1.38/1000 catheter days) and 2% CHG and 70% IPA wipes (zero events; *p* = 0.637) [41]. However, additional randomized controlled trials are needed to confirm these findings and to assess other commonly used types and concentrations of disinfectant wipes.

Several studies have demonstrated the efficacy of the “scrub the hub” protocol at reducing bacterial contamination [2, 42–45], as summarized in Table 3.

#### 3.2.2. Flushing-Based Prevention Strategies

Flushing constitutes an essential part of best practice protocol during line access, given that every access increases the risk of infection. Flushing is the process by which fluids, medications, and blood are moved out of a vascular access device (VAD) into the bloodstream, preventing substance deposition in the catheter lumen, which decreases biofilm formation and subsequently decreases the risk of CRBSI [8, 46]. The Infusion Standards of Practice advocate flushing before and after medication administration to maintain patency and to reduce the risk of drug incompatibility, occlusions, and CRBSI [4].

Catheter occlusions are associated with an increased risk of CRIs and may necessitate catheter removal or replacement, causing an interruption to patient treatment [47, 48]. Although flushing has no impact on mechanical occlusions, it has been strongly associated with the prevention of catheter occlusion caused by intraluminal sources, such as incompatible medications or the formation of a fibrin sheath [8].

It is also important to consider the type of syringe used during the flushing process as manually filled syringes may increase contamination and risk of CRI [49]. For example, a multivariate analysis indicated a significant reduction in CRBSI incidence with the use of a prefilled saline syringe versus a manually filled syringe (odds ratio [OR]: 0.40; *p* = 0.019) [49]. Similarly, another study found a significantly decreased CLABSI rate of 1.9/1000 per catheter days in the prefilled syringe arm versus 10.1/1000 catheter days in the manually filled syringe control arm (*p* < 0.05). Although not statistically significant, the study found a numerically lower rate of occlusions in the prefilled syringe group at 1.9/1000 per catheter days compared with 5.6/1000 per catheter days in the control (*p* > 0.05) [48] (Table 4).

#### 3.2.3. Locking-Based Prevention Strategies

Locking is defined as the installation of a solution (saline, heparin, or antimicrobial) into a VAD while the catheter is not in use [8]. Locking reduces the risk of occlusion and microorganism adhesion, which may lead to the formation of a biofilm and subsequent BSI, and is commonly used in infrequently accessed lines [4, 8].

Antimicrobial locking solutions (e.g., gentamicin) reduce the risk of CRBSI, with one meta-analysis of 23 studies indicating a 69% reduction in CLABSI rates compared to heparin (relative risk [RR]: 0.31) [53]. Although most studies indicate a beneficial effect of antimicrobial locking solutions for CRI prevention, this must be balanced by the potential for adverse effects or resistance associated with an antimicrobial agent [39]. Instead, saline or heparin is typically used to lock VADs, with antimicrobial locking solutions only recommended for use in patients with a history of multiple CRBSIs or with long-term CVADs [4]. A 2018 Cochrane review of 11 studies reported that there is no clear difference between heparin and saline locking solutions as they relate to the prevention of CRBSI (RR: 0.66) [54, 55]. However, in patients with long-term hemodialysis catheters, sodium–citrate locking solution (4%) resulted in fewer CRIs when compared with heparin [56].

#### 3.2.4. Disinfectant Cap–Based Prevention Strategies

Alcohol-containing passive hub disinfection caps are increasingly being used as a CRBSI prevention strategy based on evidence showing their efficacy in decreasing CLABSI risk [2, 9, 10]. Disinfection caps are alcohol-impregnated covers placed upon access ports when not in use, creating a physical barrier to prevent bacterial colonization of catheter hubs while passively disinfecting the port [57].

Studies have reported infection reduction (42%–86%) with the introduction of passive disinfectant caps [2, 10], with one study reporting a decrease in CLABSI rates from 1.43/1000 catheter days to 0.69/1000 [9] (Table 5). When compared to a standard (not impregnated with alcohol) NC cap, a 2021 trial found that the infection risk was 13.7 times lower in the group that used an IPA cap [58].

Alcohol caps may improve compliance by passive disinfection and consequently improve catheter hub decontamination compared to manual scrubbing [2]. Indeed, a meta-analysis of 10 studies confirmed that alcohol-impregnated caps were associated with significantly fewer CRIs than 70% alcohol wipes (risk ratio: 0.43; 95% CI: 0.28–0.65) [40]. A multicenter study found that the implementation of a disinfectant cap ($2.07 per patient per day) prevented 21 infections and four deaths annually, yielding annual savings of $390,617 [9]. Similarly, another study found that disinfectant cap use resulted in 50% fewer CLABSIs in the first 21 months after introduction, resulting in estimated net savings of $464,440 annually [59].

However, there remains the potential for user error, such as cross-contamination between cap implementations or contamination of the catheter between cap removal and line access. The addition of active measures, such as scrubbing, may help to mitigate this risk of cross-contamination.

#### 3.2.5. Bundled Prevention Strategies

Prevention methods are combined into “bundles” to reduce the risk of bacterial contamination of catheter hubs and CRBSI risk by improving staff compliance through best practices [1, 27, 60]. PIVC insertion and maintenance bundles are a set of up to five evidence-based practices, combined to simplify extensive guidelines for catheter care [61]. A systematic literature review (SLR) investigating the efficacy of PIVC bundles in preventing complications and infections found that 12 of 13 studies reported reductions in BSI rates upon bundle implementation [61]. Seven of these studies reported a relative reduction in BSI rates ranging from 19% to 81% [61]. Although the quality of the included studies was ranked between low and fair, a separate meta-analysis corroborated these findings, showing that central line bundles (CLBs) decreased infection incidence significantly (incidence risk ratio [IRR]: 0.44; 95% CI: 0.39–0.50; *p* < 0.0001; *I*^2^ = 89%) [30]. The implementation of CLBs decreased CLABSI rates across all types of ICU studies (i.e., adult, pediatric, and neonatal) [30].

A meta-analysis of 59 studies found that the reduction in CLABSI was 56% upon bundle implementation (RR: 0.44; 95% CI: 0.39–0.5). In contrast, studies that included compliance monitoring as part of bundle implementation saw a 61% reduction in CLABSI, although considerable heterogeneity was evident in this group [62]. Evidently, compliance with disinfection protocols and best practices plays a significant role in CRBSI risk reduction.

### 3.3. Real-World Compliance With CLABSI Prevention Recommendations and Current Challenges

Despite several guidelines recommending the use of the “scrub the hub” and other infection prevention protocols such as flushing (e.g., the SASH protocol) [5, 6], real-world observations suggest that there are significant gaps in catheter care, maintenance, and adherence to best practices, a major risk factor for the development of CRI and other complications, such as occlusion and phlebitis [47, 48, 63, 64]. Numerous studies have documented low catheter care compliance rates, ranging from 10% for scrubbing to 85% for flushing (Table 6) [2, 11, 12].

Various studies attribute noncompliance to lack of universal protocols, excessive nursing workloads, or nurses omission of items needed for flushing, which includes alcohol wipes [2, 10, 11]. A 2019 study reported a compliance rate of just 52% for scrubbing ≥ 5 s [67], and a national survey of hospitals in Thailand reported that 49% of healthcare workers did not disinfect the connectors or hubs at all prior to access [12]. Adherence to hub disinfection and proper maintenance protocols is particularly critical as increased compliance is associated with a reduced risk in CRI [2]. Simplifying extensive guidelines, protocols, and policies (e.g., care bundles) improves compliance and reduces infection risk, particularly when paired with compliance monitoring (Table 6) [61, 68].

The Institute for Safe Medication Practices (ISMP) suggests that educational strategies and policies are the easiest to implement, although they rank among the least effective interventions (i.e., a low-leverage strategy based on human reliability) [69]. In contrast, “forcing functions,” which are safety design features that necessitate actions occurring in the same way each time, are the most effective type of intervention as they can eliminate the risk of error (i.e., a high leverage strategy based on system reliability), although they are more complex to introduce [69]. The ISMP notes that strategies that have the greatest impact are those that facilitate practitioners performing their jobs correctly [69]. Furthermore, the ISMP recommends that numerous high-leverage strategies be layered together to create a robust safety system. Indeed, a meta-analysis of 41 studies indicated that quality improvement interventions reduce CLABSI rates in ICUs (OR: 0.39; 95% CI: 0.33–0.46; *p* < 0.001) [27] and found that this decrease was more prominent in studies implementing checklists or bundles (*p* = 0.03) as opposed to educational strategies [27].

#### 3.3.1. Impact of COVID-19 on CLABSI Incidence and Evolving Prevention Strategies

Recent years have seen high rates of staff turnover and nursing shortages in healthcare systems across the United States [63]. A lack of experienced nurses, combined with the need for increasingly complex care, has created disparity between workforce experience and patients' complexity, termed the experience-complexity gap [70]. This gap has widened over recent years due to a steady rate of retirements among experienced nurses and a concomitant increase in newly trained nurses [70]. The COVID-19 pandemic further exacerbated the gap and strain on hospital resources, leading to healthcare worker burnout, higher patient nurse-to-patient ratios, neglect of infection–prevention measures, and inconsistencies in patient care [1, 70].

During the early phase of the pandemic, numerous studies reported elevated CLABSI rates in both COVID-19 and non-COVID-19 patients globally [71–76]. An analysis of 78 hospitals in the United States reported a 51% increase in CLABSI rates during the first 6 months of the pandemic, rising from 0.56 to 0.85 per 1000 line days (*p* < 0.001) [63]. Similarly, the National Healthcare Safety Network (NHSN) found a 47% increase in CLABSI rates from Q4 of 2019 to Q4 of 2020 across all location types [77]. Overall, a 24% increase in CLABSI rates was reported between 2019 and 2020, with the greatest increase seen in ICUs (50%) [78]. CLABSI rates remained elevated in 2021, with an overall 7% increase between 2020 and 2021 and a 10% increase in ICUs [79]. Notably, results from a 2021 national US survey suggest that this increase occurred despite near-universal use of CHG for site antisepsis and maximum sterile barrier precautions [80]. Staffing pressures were a likely contributing factor for this increase [1, 63], with US data showing that a staff shortage of 10% of days per month was associated with two additional CLABSIs per 10,000 central line days during July 2020 to June 2021 [81]. CLABSI rates began to decrease during 2022 and 2023 [82, 83], although a Spanish study found that rates failed to return to prepandemic levels, which was attributed to worker fatigue, decreased training, and greater care load [83],

Results from a Canadian quality improvement initiative show that increased training and use of alcohol-impregnated caps, among other measures, resulted in a 51% decrease in CLABSI rates during December 2021 to May 2022 (vs. July 2019 to February 2020) [84]. Similarly, an analysis of the US Premier Healthcare Database found that adding disinfecting caps was associated with a 73% decrease in CLABSI rates (0.3% vs. 1.1%; *p* = 0.0013) relative to scrubbing alone during January 2020 to September 2020 [85].

### 3.4. Comparison of CLABSI Prevention Methods and Recommendations for the Post-COVID-19 Era

Overall, there is limited rigorous comparative evidence evaluating the relative benefit of different CLABSI prevention methods, as studies often either focus on individual methods or on bundled approaches. Further, the relative ubiquity of flushing in clinical practice may limit its relevance for comparisons with other prevention methods. To address these limitations, in our practice, we routinely combine multiple prevention methods, including scrubbing, flushing, and disinfection caps, as well as offering recurring training with system monitoring of practice behaviors. This combined approach is necessary to address persistent clinical workforce challenges in the post-COVID-19 era, including an ongoing decrease in overall knowledge and proficiency with CLABSI prevention methods. Still, additional high-quality comparative research is needed to identify which individual prevention strategies or combinations are most efficacious, as well as to assess their overall feasibility in the post-COVID-19 era.

## 4. Conclusions

Although “scrub the hub” and flushing steps are recommended in clinical practice guidelines and are key components of widely used protocols such as SASH, there is great variability in practice and real-world compliance with these methods. Excessive nursing workloads, lack of universal protocols, and complexity of multistep protocols, which often require the use of multiple products at the bedside, all result in poor compliance. The emergence of the COVID-19 pandemic further exacerbated these challenges, reversing the previous trend towards improved CLABSI rates. Further, evidence suggests that CLABSI rates have not yet fully return to prepandemic levels due in part to ongoing staff workload and educational challenges. Although implementing policies and education alone are weak interventions, innovative approaches for simplifying protocols and “forcing function” may be more effective at increasing compliance, changing behavior, and mitigating risk. As there is currently limited robust evidence comparing the relative efficacy of individual CLABSI prevention methods, in our practice, we routinely use disinfection caps in addition to standard scrubbing and flushing, alongside increased training and monitoring procedures. Additional studies are needed to assess which individual or combination prevention strategies are most efficacious, as well as their feasibility during ongoing staffing challenges in the post-COVID-19 era.

## Figures and Tables

**Table 1:** Summary of CRBSI economic burden studies and economic analyses of quality improvement interventions.

**Citation**	**Study type/details**	**Key findings**
Yu et al.[33]	Retrospective observational study of patients in 41 US acute-care hospitals (*n*CLABSI = 403; *n*non‐BSI controls = 1745)	• In case-matched analyses vs. controls without a BSI, the mean LOS was increased by 16.8 days for patients with a CLABSI • Mean hospital costs were increased by $49,400 in patients with a CLABSI vs. controls for all admission types and by $70,407 for ICU admissions
Ista et al. [30]	Systematic review and meta-analysis to quantify the effectiveness of central line bundles (search period: 1990–2015; *n* = 96 studies)	• Cost savings for CLABSI prevention were reported in 12 studies (United States: 11 studies; New Zealand: one study) • The estimated median cost per prevented CLABSI event was $42,609 (IQR $19,000–$46,739)
Rangel [34]	Quality improvement study at a 36-bed, US urban ICU (analysis period: 2019–Q1 2021)	• The estimated cost of the quality improvement initiative was $10,200 over 17 weeks to increase nurse compliance with central line dressing changes ($100/h × 2 *h*/week × 17 weeks for *three* nurses)
Nuckols et al. [35]	Systematic review of quality improvement initiatives for CLABSI/CRBSI prevention at acute-care hospitals (search period: 2004–2016; *n* = 15 studies)	• The median cost of quality improvement initiatives was $270,000 per hospital over 3 years, with costs reaching $500,000–$750,000 in certain studies

Abbreviations: BSI, bloodstream infection; CLABSI, central line–associated bloodstream infection; CRBSI, catheter-related bloodstream infection; ICU, intensive care unit; IQR, interquartile range.

**Table 2:** Overview of guidelines on scrubbing.

**Guidelines/agency**	**Key recommendations**
INS [4]	• Perform active disinfection by a vigorous mechanical scrub using a flat swab pad containing 70% isopropyl alcohol or alcohol-based chlorhexidine suitable for use with medical devices
CDC [5]	• Scrub the NC access port with CHG, povidone iodine, an iodophor or 70% alcohol, and access the port only with sterile devices
The Joint Commission [23]	• Disinfect catheter hub and stopcocks with friction using approved antiseptic swab, for example, IPA, ethyl/ethanol alcohol, and iodophors • Scrub every port on the system
Agency for Healthcare Research and Quality [38]	• Performing insertion with the central line bundle and scrubbing the hub with an appropriate antiseptic are Category 1A recommendations as critical components of CLABSI prevention programs
SHEA/IDSA [6]	• Disinfect catheter hubs, NCs, and injection ports with an alcoholic chlorhexidine preparation or 70% isopropyl alcohol. Vigorous mechanical friction should be applied for a minimum of 5 s • Compliance with guidelines should be monitored

Abbreviations: CDC, center for disease control; CHG, chlorhexidine gluconate; IDSA, Infectious Diseases Society of America; INS, Infusion Nurses Society; IPA, isopropyl alcohol; NC, needleless connector; SHEA, Society for Healthcare Epidemiology of America; SLR, systematic literature review.

**Table 3:** Studies evaluating the use of “scrub the hub”–based protocols for reducing the risk of bacterial contamination/infection.

**Citation**	**Study type/details**	**“Scrub the hub” protocol**	**Key findings**
Luders et al. [43]	Quasiexperimental study in hemodialysis unit	Only described as “scrub the hub”	• Over a period of 1430 days, only two CRBSIs were observed. CRBSI rates fell from 1.1/1000 catheter days to 0.08 over the study period.
Devrim et al. [44]	Prospective pre- and postinterventional study	Scrubbing the NC for 15 s with 70% alcohol	• Found to be successful at eliminating the colonization of the surface of needleless connectors. Colonization decreased from 20% of NCs to 0%.
Moureau and Flynn [2]	SLR	Scrubbing NC with 70% alcohol	• Disinfection with 70% isopropyl alcohol eliminated all microorganisms.
Rupp et al. [45]	Prospective observational clinical survey and laboratory assessment	Scrubbing needleless intravascular connector valves with 70% isopropyl alcohol for 0,5,10,15, or 30 s	• In a clinical setting, 363 connector valves were sampled. Of nondisinfected valves, 66.7% displayed bacterial contamination. After a 5 s scrub, only one (1.4%) of 71 indicated microbial growth (*p* < 0.005). • In the laboratory, a 5-s scrub of connector valves yielded sterile cultures (*p* < 0.001).

Abbreviations: CRBSI, catheter-related bloodstream infection; NC, needleless connector; SLR, systematic literature review.

**Table 4:** Studies evaluating flushing protocols with respect to bacterial contamination/infection risk.

**Citation**	**Study type/details**	**Flushing protocol**	**Key findings**
Zhong et al. [50]	SLR	Flushing with normal saline vs. heparin	• Flushing with NS was as effective as heparin solution in preventing CRBSI (*n* = 1630; RR, 0.84; 95%CI = 0.11–6.71; *p* = 0.871).
Ribeiro et al. [51]	Integrative literature review	Flushing with prefilled syringes	• Use of prefilled syringes significantly reduced PVC failures and complication occurrence, increased the catheter dwell time, and reduced related costs. • Advanced degrees of phlebitis were associated with incorrect PVC flushing protocol. • A comparison of manual filling vs. prefilled syringes indicated a 77% and 62% reduction in BSI occurrence associated with the catheter and occlusion, respectively.
Gerçeker et al. [48]	Prospective, randomized study	Manually prepared syringes vs. single-used prefilled syringes	• In the intervention group, CLABSI rate was 1.9/1000 per catheter days, and in the control group, CLABSI rate was 10.1/1000 per catheter days. In the intervention group, occlusion rate was 1.9/1000 per catheter days, and in the control group, occlusion rate was 5.6/1000 per catheter days.
Saliba et al. [52]	Quasiexperimental study	Manually prepared syringes vs. single-used prefilled syringes	• The use of prefilled syringes significantly decreased peripheral venous catheter failure rates vs. manually filled syringes (57% vs. 43.4%; *p* < 0.001).

Abbreviations: BSI, bloodstream infection; CI, confidence interval; CLABSI, central line–associated bloodstream infection; CRI, catheter-related infection; NC, needleless connector; NS, normal saline; PVC, peripheral venous catheter; RR, relative risk; SLR, systematic literature review.

**Table 5:** Studies evaluating disinfectant caps with respect to bacterial contamination/infection risk.

**Citation**	**Study details**	**Prevention protocol(s)**	**Key findings**
Wright et al. [9]	Multiphase, prospective, quasiexperimental study	• 70% isopropyl alcohol caps	• CLABSI rates declined from 1.43/1000 line days to 0.69 with the introduction of the cap. • Removal of the cap from practice caused a subsequent increase in CLABSI rates to 1.31/1000 line days.
Moureau and Flynn [2]	SLR	• Passive disinfection caps	• Studies have reported significant infection reduction when passive disinfectant caps are used (48%–86% reduction). • Decreased from 1.682/1000 catheter days to 0.6461/1000 after introduction of the cap.
Öğülmen and Ates [58]	RCT	• Isopropyl alcohol cap on needleless connector	• The infection risk was found to be 13.7 lower in the intervention group vs. the control group (standard catheter caps).
Greene [10]	Review	• Isopropyl alcohol cap	• After switching from manual disinfection to caps on all DNCCs, CLABSI rates decreased by 42% (*p* = 0.004).

Abbreviations: CLABSI, central line–associated bloodstream infection; DNCC, disinfectable needleless connector; PIVC, peripheral intravenous catheter; RCT, randomized controlled trial; SLR, systematic literature review.

**Table 6:** Summary of studies reporting compliance with CRBSI prevention protocols.

**Citation**	**Study details**	**Prevention protocol(s)**	**Key findings**
Santos-Costa et al. [11]	Observational, prospective study	• Scrubbing the hub prior to use with either CHG or 70% isopropyl alcohol • 0.9% sodium chloride flush before and after each use	• Overall, in 54% of the observations made, nurses scrubbed the NC before each use • Postinsertion flushing was performed only 67% of the time, with saline volumes varying • Catheter flushing before and after drug administration was performed 85% of the time • Nurses often overlook flushing due to high workloads, lack of recent training, and omission of material for flushing. Recommends the use of prefilled syringes
Moureau and Flynn [2]	SLR	• Scrub the hub; hub disinfection	• Compliance to disinfection measures before NC access was as low as 10% • When evaluating 5877 healthcare professionals, a study documented compliance with hub disinfection to be 38.7%
Parreira et al. [65]	Cross-sectional study	• Flushing after PIVC insertion, before drug administration, between drug administration and after last administration	• 73.4% responded that they flush on these four occasions
Seddon et al. [66]	CLABSI quality improvement study	• Quality improvement initiative implemented, including central line insertion/maintenance checklists, training, and monitoring	• Compliance with maintenance measures improved from 64% to 85%, while CLABSI rates decreased from a mean of 2.3/month to 0.56/month • Hospital-wide CLABSI rates decreased from 7.04/1000-line days to 1.37/1000

Abbreviations: CHG, chlorhexidine gluconate; CLABSI, central line–associated bloodstream infection; NC, needleless connector; PIVC, peripheral intravenous catheter; SLR, systematic literature review.

## Data Availability

Data sharing is not applicable to this article as no new data were created or analyzed in this study.
