# Supplementary material for: Prevention of Catheter-Related Infections and Complications: A Narrative Literature Review of Vascular Care and Maintenance
Source: Int J Vasc Med. 2025 Aug 1;2025:1427129. doi: 10.1155/ijvm/1427129 (PMC12334285; doi:10.1155/ijvm/1427129)
Supplement: Supporting Information — Additional supporting information can be found online in the Supporting Information section. Appendix: Search strategy. [file 1427129.f1.docx]

**Prevention of Catheter-Related Infections and Complications: A Narrative Literature Review of Vascular Care and Maintenance**

Dr Nathan T. Gilmore, M.D., M.B.A.^1,*^, Dr Terrence Metz, M.D, MEng.^2^

^1^Department of Critical Care, Hoag Hospital, Newport Beach, CA, USA, [ngilmore@npccp.com](mailto:ngilmore@npccp.com)

^2^ Department of Radiology, Division of Interventional Radiology, Corewell Health William Beaumont University Hospital, Royal Oak, MI, USA, [terrencemetz@gmail.com](mailto:terrencemetz@gmail.com)

* Corresponding Author

# **Search Strategy**

**Electronic Databases and Search Period**:

- EBM Reviews - Cochrane Central Register of Controlled Trials <August 2022>
- EBM Reviews - Cochrane Database of Systematic Reviews <2005 to September 21, 2022>
- Embase <1974 to 2022 September 22>
- Ovid MEDLINE(R) and Epub Ahead of Print, In-Process, In-Data-Review & Other Non-Indexed Citations and Daily <1946 to September 22, 2022>

**Literature Search Strategy:**

- The search strategy was developed and tested through an iterative process by an experienced medical information specialist in consultation with both authors.
  - Utilized a combination of controlled vocabulary (e.g., “Catheters”, “Catheterization”, “Catheter-Related Infections”, “Disinfection”, “Decontamination”) and various permutations of relevant keywords (e.g., “vascular access device”, “needleless connector”, “access port”).
- No restrictions were applied on the date or publication types, with the exception of conference abstracts in Embase.
- Database searches were executed on September 23^rd^, 2022.
- Titles and abstracts were screened in DistillerSR by both authors, followed by a review of full text of all citations identified as potentially relevant.
- Studies solely based on animal evidence were excluded; no restrictions were applied for human evidence. Only articles in the English language were included.

**Search Terms for the CLABSI Prevention Search**:

1 exp *Catheters/ (69869)

2 exp *Catheterization/ (185732)

3 (catheter* or microcatheter* or micro-catheter* or cannula or cannulae).ti,kw,kf. (218297)

4 (catheter* or microcatheter* or micro-catheter* or cannula or cannulae).ab. /freq=2 (231883)

5 vascular access device?.ti,kw,kf. (1105)

6 vascular access device?.ab. /freq=2 (217)

7 ((VAD or VADs) adj10 (vascular* or veno*)).ti,kw,kf. (145)

8 ((VAD or VADs) adj10 (vascular* or veno*)).ab. /freq=2 (679)

9 ((CVC or PVC) adj10 (vascular* or veno*)).ti,kw,kf. (367)

10 ((CVC or PVC) adj10 (vascular* or veno*)).ab. /freq=2 (1212)

11 (PICC or PICCs or PIVC or PIVCs).ti,kw,kf. (2174)

12 ((PICC or PICCs or PIVC or PIVCs) adj5 peripheral*).ab. (4350)

13 ((needleless or needle-less or needlefree or needle-free) adj3 (access* or connect*)).ti,kw,kf. (331)

14 access port?.ti,kw,kf. (1182)

15 access port?.ab. /freq=2 (455)

16 ((VAD or VADs or CVC or PVC or PICC or PICCs or PIVC or PIVCs or central line or central lines) adj3 (cap or caps or connector? or hub or hubs or port or ports)).ti,kw,kf. (96)

17 or/1-16 [CATHETERS, INCLUDING CONNECTORS, HUBS] (455924)

18 (care adj (bundl* or practice?)).tw,kw,kf. (45481)

19 ((cannula or cannulae or catheter* or CVC or PVC or VAD or VADs or PICC or PICCs or PIVC or PIVCs or central line or central lines) adj3 (bundle? or care)).tw,kw,kf. (6299)

20 *Catheter-Related Infections/pc [Prevention & Control] (2827)

21 exp *Cross Infection/pc [Prevention & Control] (21996)

22 *Decontamination/ (4642)

23 *Disinfection/ (22154)

24 *Disinfectants/ (17364)

25 *Equipment Contamination/pc [Prevention & Control] (2222)

26 *Infection Control/ (45578)

27 exp *Antisepsis/ (4238)

28 exp *Sepsis/pc [Prevention & Control] (8151)

29 ((CLABSI or CLABSIs or CRBSI or CRBSIs or HAI or HAIs or bloodstream infection? or blood-stream infection? or septic?emia* or sepsis) adj3 (avert* or avoid* or prevent* or reduc* or stop*)).ti,kw,kf. (2623)

30 ((CLABSI or CLABSIs or CRBSI or CRBSIs or HAI or HAIs or bloodstream infection? or blood-stream infection? or septic?emia* or sepsis) adj3 (avert* or avoid* or prevent* or reduc* or stop*)).ab. /freq=2 (2278)

31 ((cannula or cannulae or catheter* or CVC or PVC or VAD or VADs or PICC or PICCs or PIVC or PIVCs or central lines) adj3 (antiseptic* or anti-septic* or clean* or decontaminat* or de-contaminat* or disinfect* or flush* or lock* or sanitis* or sanitiz* or scrub* or sterili* or swab*)).ti,ab,kw,kf. (9883)

32 ((cap or caps or hub or hubs or port or ports or connector?) adj3 (antiseptic* or anti-septic* or clean* or decontaminat* or de-contaminat* or disinfect* or flush* or lock* or sanitis* or sanitiz* or scrub* or sterili* or swab*)).ti,ab,kw,kf. (1280)

33 (device? adj3 (antiseptic* or anti-septic* or clean* or decontaminat* or de-contaminat* or disinfect* or flush* or sanitis* or sanitiz* or scrub* or sterili* or swab*)).ti,ab,kw,kf. (4511)

34 ((cap or caps or hub or hubs or port or ports or connector?) adj3 (contaminat* or infect*) adj3 (avert* or avoid* or prevent* or reduc* or stop*)).ti,ab,kw,kf. (109)

35 ((device? or method or methods or practice?) adj3 (contaminat* or infect*) adj3 (avert* or avoid* or prevent* or reduc* or stop*)).ti,ab,kw,kf. (4855)

36 ((prefil* or pre-fill*) adj saline).ti,ab,kw,kf. (40)

37 flush*.ab. /freq=3 (8037)

38 (PosiFlush* or SiteScrub* or SwabFlush*).mp. (28)

39 or/18-38 [CLABSI PREVENTION] (192274)

40 17 and 39 [CATHETERS/HUBS - CLABSI PREVENTION] (18190)

41 exp Animals/ not Humans/ [ANIMAL STUDIES ONLY - REMOVE - MEDLINE] (16523183)

42 40 not 41 [ANIMAL-ONLY REMOVED] (15409)

43 (address or autobiography or bibliography or biography or comment or dictionary or directory or editorial or "expression of concern" or festschrift or historical article or interactive tutorial or lecture or legal case or legislation or news or newspaper article or patient education handout or personal narrative or portrait or video-audio media or webcast or (letter not (letter and randomized controlled trial))).pt. [Opinion publications - Remove -MEDLINE] (4728768)

44 42 not 43 [OPINION PIECES REMOVED] (14639)

45 limit 44 to yr="2012-current" [DATE LIMIT APPLIED] (6818)

46 limit 45 to english [LANGUAGE LIMIT APPLIED] (6578)

47 Systematic Review.pt. (216463)

48 exp Systematic Reviews as Topic/ (39001)

49 Meta Analysis.pt. (168344)

50 exp Meta-Analysis as Topic/ (76441)

51 (meta-analy* or metanaly* or metaanaly* or met analy* or integrative research or integrative review* or integrative overview* or research integration or research overview* or collaborative review*).tw,kw,kf. (611873)

52 (systematic review* or systematic overview* or evidence-based review* or evidence-based overview* or (evidence adj3 (review* or overview*)) or meta-review* or meta-overview* or meta-synthes* or mapping review? or rapid review* or "review of reviews" or scoping review? or umbrella review? or technology assessment* or HTA or HTAs).tw,kw,kf. (762718)

53 exp Technology Assessment, Biomedical/ (28198)

54 (cochrane or health technology assessment or evidence report or systematic reviews).jw. (67603)

55 Network Meta-Analysis/ or ((network adj (MA or MAs)) or (NMA or NMAs or MTC or MTCs or MAIC or MAICs) or indirect* compar* or (indirect treatment* adj1 compar*) or (mixed treatment* adj1 compar*) or (multiple treatment* adj1 compar*) or (multi-treatment* adj1 compar*) or simultaneous* compar* or mixed comparison?).tw,kw,kf. (41068)

56 or/47-55 [REVIEW FILTER] (1214293)

57 46 and 56 [REVIEWS] (490)

58 (controlled clinical trial or randomized controlled trial or pragmatic clinical trial or equivalence trial).pt. (1321239)

59 "Clinical Trials as Topic"/ (323834)

60 exp "Controlled Clinical Trials as Topic"/ (419899)

61 (randomi#ed or randomi#ation? or randomly or RCT or placebo*).tw,kw,kf. (4070626)

62 ((singl* or doubl* or trebl* or tripl*) adj (mask* or blind* or dumm*)).tw,kw,kf. (803891)

63 trial.ti. (1023286)

64 or/58-63 [RCT FILTER - BEST BALANCE BASED ON COCHRANE HSSS] (5051599)

65 46 and 64 [RCTs] (1679)

66 controlled clinical trial.pt. (188301)

67 Controlled Clinical Trial/ or Controlled Clinical Trials as Topic/ (578845)

68 (control* adj2 trial).tw,kw,kf. (1145822)

69 Non-Randomized Controlled Trials as Topic/ (13661)

70 (nonrandom* or non-random* or quasi-random* or quasi-experiment*).tw,kw,kf. (180746)

71 (nRCT or non-RCT).tw,kw,kf. (1440)

72 Controlled Before-After Studies/ (224849)

73 (control* adj3 ("before and after" or "before after")).tw,kw,kf. (13817)

74 Interrupted Time Series Analysis/ (218127)

75 time series.tw,kf. (90839)

76 (pre- adj5 post-).tw,kw,kf. (374705)

77 ((pretest adj5 posttest) or (pre-test adj5 post-test)).tw,kw,kf. (36013)

78 Historically Controlled Study/ (235256)

79 (control* adj2 study).tw,kw,kf. (1040337)

80 Control Groups/ (112484)

81 (control* adj2 group?).tw,kw,kf. (1699116)

82 trial.ti. (1023286)

83 or/66-82 [nRCT FILTER] (4768048)

84 46 and 83 [nRCTs] (1649)

85 exp Cohort Studies/ (3457916)

86 cohort?.tw,kw,kf. (2192777)

87 Retrospective Studies/ (2101942)

88 (longitudinal or prospective or retrospective).tw,kw,kf. (4263804)

89 ((followup or follow-up) adj (study or studies)).tw,kw,kf. (139979)

90 Observational study.pt. (134185)

91 (observation$2 adj (study or studies)).tw,kw,kf. (399218)

92 ((population or population-based) adj (study or studies or analys#s)).tw,kw,kf. (57196)

93 ((multidimensional or multi-dimensional) adj (study or studies)).tw,kw,kf. (323)

94 Comparative Study.pt. (2082604)

95 ((comparative or comparison) adj (study or studies)).tw,kw,kf. (330239)

96 exp Case-Control Studies/ (1581939)

97 ((case-control* or case-based or case-comparison or case-compeer or case-referrent or case-referent) adj3 (study or studies)).tw,kw,kf. (319611)

98 Cross-Sectional Studies/ (823804)

99 (crosssection* or cross-section*).tw,kw,kf. (1200142)

100 (real-world or RWE).tw,kw,kf. (189757)

101 ("single arm" adj2 (stud$ or design?)).tw,kw,kf. (13235)

102 or/85-101 [OBSERVATIONAL STUDY FILTER, INCLUDING SINGLE-ARM STUDIES] (10852327)

103 46 and 102 [OBSERVATIONAL STUDIES] (2689)

104 exp Guidelines as Topic/ or exp Clinical Protocols/ or (Guideline or Practice Guideline or Consensus Development Conference or Consensus Development Conference, NIH).pt. or (consensus or guideline$ or standards or recommendation$).ti. or (expert consensus or consensus statement$ or consensus conference$ or practice parameter$ or position statement$ or policy statement$ or CPG or CPGs).ti,kf. (1337162)

105 46 and 104 [CPGs] (535)

106 (best practice? or evidence-based).ti,kw,kf. (116643)

107 (policy or policies).ti,kw,kf. (191066)

108 106 or 107 [BEST PRACTICE, POLICY FILTER] (304424)

109 46 and 108 [BEST PRACTICES, POLICIES] (195)

110 57 or 65 or 84 or 103 or 105 or 109 (4329)

111 110 use ppez [MEDLINE RECORDS] (1863)

112 exp *catheter/ (69456)

113 exp *catherization/ (58318)

114 (catheter* or microcatheter* or micro-catheter* or cannula or cannulae).ti,kw,kf. (218297)

115 (catheter* or microcatheter* or micro-catheter* or cannula or cannulae).ab. /freq=3 (130110)

116 vascular access device?.ti,kw,kf. (1105)

117 vascular access device?.ab. /freq=3 (74)

118 ((VAD or VADs) adj10 (vascular* or veno*)).ti,kw,kf. (145)

119 ((VAD or VADs) adj10 (vascular* or veno*)).ab. /freq=3 (165)

120 ((CVC or PVC) adj10 (vascular* or veno*)).ti,kw,kf. (367)

121 ((CVC or PVC) adj10 (vascular* or veno*)).ab. /freq=3 (310)

122 (PICC or PICCs or PIVC or PIVCs).ti,kw,kf. (2174)

123 ((PICC or PICCs or PIVC or PIVCs) adj5 peripheral*).ab. (4350)

124 needleless connector/ (63)

125 ((needleless or needle-less or needlefree or needle-free) adj3 (access* or connect*)).ti,kw,kf. (331)

126 access port?.ti,kw,kf. (1182)

127 access port?.ab. /freq=3 (150)

128 ((VAD or VADs or CVC or PVC or PICC or PICCs or PIVC or PIVCs or central line or central lines) adj3 (cap or caps or connector? or hub or hubs or port or ports)).ti,kw,kf. (96)

129 or/112-128 [CATHETERS, INCLUDING CONNECTORS, HUBS] (307186)

130 (care adj (bundl* or practice?)).ti,kw,kf. (9752)

131 (care adj (bundl* or practice?)).ab. /freq=2 (7886)

132 ((cannula or cannulae or catheter* or CVC or PVC or VAD or VADs or PICC or PICCs or PIVC or PIVCs or central line or central lines) adj3 (bundle? or care)).ti,kw,kf. (1615)

133 ((cannula or cannulae or catheter* or CVC or PVC or VAD or VADs or PICC or PICCs or PIVC or PIVCs or central line or central lines) adj3 (bundle? or care)).ab. /freq=2 (1192)

134 *catheter infection/pc [Prevention & Control] (1981)

135 *cross infection/pc [Prevention & Control] (21185)

136 *decontamination/ (4642)

137 *disinfection/ (22154)

138 exp *disinfectant agent/ (197113)

139 *medical device contamination/pc [Prevention & Control] (22)

140 *infection control/ (45578)

141 *asepsis/ (2722)

142 *antisepsis/ (3832)

143 exp *sepsis/pc [Prevention & Control] (8151)

144 ((CLABSI or CLABSIs or CRBSI or CRBSIs or HAI or HAIs or bloodstream infection? or blood-stream infection? or septic?emia* or sepsis) adj3 (avert* or avoid* or prevent* or reduc* or stop*)).ti,kw,kf. (2623)

145 ((CLABSI or CLABSIs or CRBSI or CRBSIs or HAI or HAIs or bloodstream infection? or blood-stream infection? or septic?emia* or sepsis) adj3 (avert* or avoid* or prevent* or reduc* or stop*)).ab. /freq=3 (790)

146 ((cannula or cannulae or catheter* or CVC or PVC or VAD or VADs or PICC or PICCs or PIVC or PIVCs or central lines) adj3 (antiseptic* or anti-septic* or clean* or decontaminat* or de-contaminat* or disinfect* or flush* or lock* or sanitis* or sanitiz* or scrub* or sterili* or swab*)).ti,ab,kw,kf. (9883)

147 ((cap or caps or hub or hubs or port or ports or connector?) adj3 (antiseptic* or anti-septic* or clean* or decontaminat* or de-contaminat* or disinfect* or flush* or lock* or sanitis* or sanitiz* or scrub* or sterili* or swab*)).ti,ab,kw,kf. (1280)

148 (device? adj3 (antiseptic* or anti-septic* or clean* or decontaminat* or de-contaminat* or disinfect* or flush* or sanitis* or sanitiz* or scrub* or sterili* or swab*)).ti,ab,kw,kf. (4511)

149 ((cap or caps or hub or hubs or port or ports or connector?) adj3 (contaminat* or infect*) adj3 (avert* or avoid* or prevent* or reduc* or stop*)).ti,ab,kw,kf. (109)

150 ((device? or method or methods or practice?) adj3 (contaminat* or infect*) adj3 (avert* or avoid* or prevent* or reduc* or stop*)).ti,ab,kw,kf. (4855)

151 ((prefil* or pre-fill*) adj saline).ti,ab,kw,kf. (40)

152 flush*.ab. /freq=3 (8037)

153 (PosiFlush* or SiteScrub* or SwabFlush*).mp. (28)

154 or/130-153 [CLABSI PREVENTION] (335585)

155 129 and 154 [CATHETERS/HUBS - CLABSI PREVENTION] (14217)

156 (exp animal/ or exp animal experimentation/ or exp animal model/ or exp animal experiment/ or nonhuman/ or exp vertebrate/) not (exp human/ or exp human experimentation/ or exp human experiment/) (12050540)

157 exp human/ or exp human experimentation/ or exp human experiment/ (45538649)

158 156 not 157 (12050540)

159 155 not 158 [ANIMAL-ONLY REMOVED] (13653)

160 (editorial or letter).pt. not (letter.pt. and randomized controlled trial/) (3788922)

161 159 not 160 [OPINION PIECES REMOVED] (12992)

162 limit 161 to yr="2012-current" [DATE LIMIT APPLIED] (6550)

163 limit 162 to english [LANGUAGE LIMIT APPLIED] (6316)

164 "systematic review"/ (578205)

165 "systematic review (topic)"/ (29892)

166 meta analysis/ (425267)

167 "meta analysis (topic)"/ (50496)

168 (meta-analy* or metanaly* or metaanaly* or met analy* or integrative research or integrative review* or integrative overview* or research integration or research overview* or collaborative review*).tw,kw,kf. (611873)

169 (systematic review* or systematic overview* or evidence-based review* or evidence-based overview* or (evidence adj3 (review* or overview*)) or meta-review* or meta-overview* or meta-synthes* or mapping review? or rapid review* or "review of reviews" or scoping review? or umbrella review? or technology assessment* or HTA or HTAs).tw,kw,kf. (762718)

170 biomedical technology assessment/ (27041)

171 (cochrane or health technology assessment or evidence report or systematic reviews).jw. (67603)

172 network meta-analysis/ or ((network adj (MA or MAs)) or (NMA or NMAs or MTC or MTCs or MAIC or MAICs) or indirect* compar* or (indirect treatment* adj1 compar*) or (mixed treatment* adj1 compar*) or (multiple treatment* adj1 compar*) or (multi-treatment* adj1 compar*) or simultaneous* compar* or mixed comparison?).tw,kw,kf. (41068)

173 or/164-172 [REVIEW FILTER] (1314071)

174 163 and 173 [REVIEWS] (515)

175 exp randomized controlled trial/ or controlled clinical trial/ (1590843)

176 clinical trial/ (1582863)

177 exp "controlled clinical trial (topic)"/ (243842)

178 (randomi#ed or randomi#ation? or randomly or RCT or placebo*).tw,kw,kf. (4070626)

179 ((singl* or doubl* or trebl* or tripl*) adj (mask* or blind* or dumm*)).tw,kw,kf. (803891)

180 trial.ti. (1023286)

181 or/175-180 [RCT FILTER - BEST BALANCE BASED ON COCHRANE HSSS] (5589407)

182 163 and 181 [RCTs] (1693)

183 controlled clinical trial/ (562287)

184 "controlled clinical trial (topic)"/ (12560)

185 (control* adj2 trial).tw,kw,kf. (1145822)

186 (nonrandom* or non-random* or quasi-random* or quasi-experiment*).tw,kw,kf. (180746)

187 (nRCT or non-RCT).tw,kw,kf. (1440)

188 (control* adj3 ("before and after" or "before after")).tw,kw,kf. (13817)

189 time series analysis/ (33944)

190 time series.tw,kf. (90839)

191 pretest posttest control group design/ (609)

192 (pre- adj5 post-).tw,kw,kf. (374705)

193 ((pretest adj5 posttest) or (pre-test adj5 post-test)).tw,kw,kf. (36013)

194 controlled study/ (9103523)

195 (control* adj2 study).tw,kw,kf. (1040337)

196 control group/ (112367)

197 (control* adj2 group?).tw,kw,kf. (1699116)

198 trial.ti. (1023286)

199 or/183-198 [nRCT FILTER] (12238517)

200 163 and 199 [nRCTs] (2313)

201 cohort analysis/ (1217620)

202 cohort?.tw,kw,kf. (2192777)

203 retrospective study/ (2371085)

204 longitudinal study/ (339016)

205 prospective study/ (1435972)

206 (longitudinal or prospective or retrospective).tw,kw,kf. (4263804)

207 follow up/ (1898086)

208 ((followup or follow-up) adj (study or studies)).tw,kw,kf. (139979)

209 observational study/ (421272)

210 (observation$2 adj (study or studies)).tw,kw,kf. (399218)

211 population research/ (125816)

212 ((population or population-based) adj (study or studies or analys#s)).tw,kw,kf. (57196)

213 ((multidimensional or multi-dimensional) adj (study or studies)).tw,kw,kf. (323)

214 exp comparative study/ (3499294)

215 ((comparative or comparison) adj (study or studies)).tw,kw,kf. (330239)

216 exp case control study/ (1581939)

217 ((case-control* or case-based or case-comparison or case-compeer or case-referrent or case-referent) adj3 (study or studies)).tw,kw,kf. (319611)

218 cross-sectional study/ (952136)

219 (crosssection* or cross-section*).tw,kw,kf. (1200142)

220 major clinical study/ (4612846)

221 (real-world or RWE).tw,kw,kf. (189757)

222 ("single arm" adj2 (stud$ or design?)).tw,kw,kf. (13235)

223 or/201-222 [OBSERVATIONAL STUDY FILTER, INCLUDING SINGLE-ARM STUDIES] (15055927)

224 163 and 223 [OBSERVATIONAL STUDIES] (3066)

225 exp practice guideline/ or (consensus or guideline$ or standards or recommendation$).ti. or (expert consensus or consensus statement$ or consensus conference$ or practice parameter$ or position statement$ or policy statement$ or CPG or CPGs).ti,kf. (1008133)

226 163 and 225 [CPGs] (387)

227 (best practice? or evidence-based).ti,kw,kf. (116643)

228 (policy or policies).ti,kw,kf. (191066)

229 227 or 228 [BEST PRACTICE, POLICY FILTER] (304424)

230 163 and 229 [BEST PRACTICES, POLICIES] (156)

231 174 or 182 or 200 or 224 or 226 or 230 (4512)

232 conference abstract.pt. (4557023)

233 222 not 232 (9395)

234 231 and 232 (1048)

235 limit 234 to yr="2020-CURRENT" (203)

236 235 use oemezd [EMBASE RECORDS] (203)

237 exp *Catheters/ (69869)

238 exp *Catheterization/ (185732)

239 (catheter* or microcatheter* or micro-catheter* or cannula or cannulae).ti,kw. (202832)

240 (catheter* or microcatheter* or micro-catheter* or cannula or cannulae).ab. /freq=2 (231883)

241 vascular access device?.ti,kw. (1057)

242 vascular access device?.ab. /freq=2 (217)

243 ((VAD or VADs) adj10 (vascular* or veno*)).ti,kw. (63)

244 ((VAD or VADs) adj10 (vascular* or veno*)).ab. /freq=2 (679)

245 ((CVC or PVC) adj10 (vascular* or veno*)).ti,kw. (209)

246 ((CVC or PVC) adj10 (vascular* or veno*)).ab. /freq=2 (1212)

247 (PICC or PICCs or PIVC or PIVCs).ti,kw. (1965)

248 ((PICC or PICCs or PIVC or PIVCs) adj5 peripheral*).ab. (4350)

249 ((needleless or needle-less or needlefree or needle-free) adj3 (access* or connect*)).ti,kw. (303)

250 access port?.ti,kw. (1086)

251 access port?.ab. /freq=2 (455)

252 ((VAD or VADs or CVC or PVC or PICC or PICCs or PIVC or PIVCs or central line or central lines) adj3 (cap or caps or connector? or hub or hubs or port or ports)).ti,ab,kw. (604)

253 or/237-252 [CATHETERS, INCLUDING CONNECTORS, HUBS] (447913)

254 (care adj (bundl* or practice?)).ti,ab,kw. (44886)

255 ((cannula or cannulae or catheter* or CVC or PVC or VAD or VADs or PICC or PICCs or PIVC or PIVCs or central line or central lines) adj3 (bundle? or care)).ti,ab,kw. (6173)

256 *Catheter-Related Infections/pc [Prevention & Control] (2827)

257 exp *Cross Infection/pc [Prevention & Control] (21996)

258 *Decontamination/ (4642)

259 *Disinfection/ (22154)

260 *Disinfectants/ (17364)

261 *Equipment Contamination/pc [Prevention & Control] (2222)

262 *Infection Control/ (45578)

263 exp *Antisepsis/ (4238)

264 exp *Sepsis/pc [Prevention & Control] (8151)

265 ((CLABSI or CLABSIs or CRBSI or CRBSIs or HAI or HAIs or bloodstream infection? or blood-stream infection? or septic?emia* or sepsis) adj3 (avert* or avoid* or prevent* or reduc* or stop*)).ti,kw. (2577)

266 ((CLABSI or CLABSIs or CRBSI or CRBSIs or HAI or HAIs or bloodstream infection? or blood-stream infection? or septic?emia* or sepsis) adj3 (avert* or avoid* or prevent* or reduc* or stop*)).ab. /freq=2 (2278)

267 ((cannula or cannulae or catheter* or CVC or PVC or VAD or VADs or PICC or PICCs or PIVC or PIVCs or central lines) adj3 (antiseptic* or anti-septic* or clean* or decontaminat* or de-contaminat* or disinfect* or flush* or lock* or sanitis* or sanitiz* or scrub* or sterili* or swab*)).ti,ab,kw. (9775)

268 ((cap or caps or hub or hubs or port or ports or connector?) adj3 (antiseptic* or anti-septic* or clean* or decontaminat* or de-contaminat* or disinfect* or flush* or lock* or sanitis* or sanitiz* or scrub* or sterili* or swab*)).ti,ab,kw. (1271)

269 (device? adj3 (antiseptic* or anti-septic* or clean* or decontaminat* or de-contaminat* or disinfect* or flush* or sanitis* or sanitiz* or scrub* or sterili* or swab*)).ti,ab,kw. (4481)

270 ((cap or caps or hub or hubs or port or ports or connector?) adj3 (contaminat* or infect*) adj3 (avert* or avoid* or prevent* or reduc* or stop*)).ti,ab,kw. (109)

271 ((device? or method or methods or practice?) adj3 (contaminat* or infect*) adj3 (avert* or avoid* or prevent* or reduc* or stop*)).ti,ab,kw. (4845)

272 ((prefil* or pre-fill*) adj saline).ti,ab,kw. (40)

273 flush*.ab. /freq=3 (8037)

274 (PosiFlush* or SiteScrub* or SwabFlush*).mp. (28)

275 or/254-274 [CLABSI PREVENTION] (191468)

276 253 and 275 [CATHETERS/HUBS - CLABSI PREVENTION] (17885)

277 (conference abstract or journal conference abstract).pt. (4751656)

278 276 not 277 [CONFERENCE ABSTRACTS REMOVED] (14967)

279 276 and 277 (2918)

280 limit 279 to yr="2020-current" (418)

281 278 or 280 [MOST RECENT 2 YRS CONFERENCE ABSTRACTS RETAINED] (15385)

282 limit 281 to yr="2012-current" [DATE LIMIT APPLIED] (7032)

283 limit 282 to english [LANGUAGE LIMIT APPLIED] (6722)

284 283 use cctr [CENTRAL RECORDS] (719)

285 (catheter* or microcatheter* or micro-catheter* or cannula or cannulae).ti,kw. (202832)

286 (catheter* or microcatheter* or micro-catheter* or cannula or cannulae).ab. /freq=2 (231883)

287 vascular access device?.ti,kw. (1057)

288 vascular access device?.ab. /freq=2 (217)

289 ((VAD or VADs) adj10 (vascular* or veno*)).ti,kw. (63)

290 ((VAD or VADs) adj10 (vascular* or veno*)).ab. /freq=2 (679)

291 ((CVC or PVC) adj10 (vascular* or veno*)).ti,kw. (209)

292 ((CVC or PVC) adj10 (vascular* or veno*)).ab. /freq=2 (1212)

293 (PICC or PICCs or PIVC or PIVCs).ti,kw. (1965)

294 ((PICC or PICCs or PIVC or PIVCs) adj5 peripheral*).ab. (4350)

295 ((needleless or needle-less or needlefree or needle-free) adj3 (access* or connect*)).ti,kw. (303)

296 access port?.ti,kw. (1086)

297 access port?.ab. /freq=2 (455)

298 ((VAD or VADs or CVC or PVC or PICC or PICCs or PIVC or PIVCs or central line or central lines) adj3 (cap or caps or connector? or hub or hubs or port or ports)).ti,ab,kw. (604)

299 or/285-298 [CATHETERS, INCLUDING CONNECTORS, HUBS] (339149)

300 (care adj (bundl* or practice?)).ti,ab,kw. (44886)

301 ((cannula or cannulae or catheter* or CVC or PVC or VAD or VADs or PICC or PICCs or PIVC or PIVCs or central line or central lines) adj3 (bundle? or care)).ti,ab,kw. (6173)

302 ((CLABSI or CLABSIs or CRBSI or CRBSIs or HAI or HAIs or bloodstream infection? or blood-stream infection? or septic?emia* or sepsis) adj3 (avert* or avoid* or prevent* or reduc* or stop*)).ti,kw. (2577)

303 ((CLABSI or CLABSIs or CRBSI or CRBSIs or HAI or HAIs or bloodstream infection? or blood-stream infection? or septic?emia* or sepsis) adj3 (avert* or avoid* or prevent* or reduc* or stop*)).ab. /freq=2 (2278)

304 ((cannula or cannulae or catheter* or CVC or PVC or VAD or VADs or PICC or PICCs or PIVC or PIVCs or central lines) adj3 (antiseptic* or anti-septic* or clean* or decontaminat* or de-contaminat* or disinfect* or flush* or lock* or sanitis* or sanitiz* or scrub* or sterili* or swab*)).ti,ab,kw. (9775)

305 ((cap or caps or hub or hubs or port or ports or connector?) adj3 (antiseptic* or anti-septic* or clean* or decontaminat* or de-contaminat* or disinfect* or flush* or lock* or sanitis* or sanitiz* or scrub* or sterili* or swab*)).ti,ab,kw. (1271)

306 (device? adj3 (antiseptic* or anti-septic* or clean* or decontaminat* or de-contaminat* or disinfect* or flush* or sanitis* or sanitiz* or scrub* or sterili* or swab*)).ti,ab,kw. (4481)

307 ((cap or caps or hub or hubs or port or ports or connector?) adj3 (contaminat* or infect*) adj3 (avert* or avoid* or prevent* or reduc* or stop*)).ti,ab,kw. (109)

308 ((device? or method or methods or practice?) adj3 (contaminat* or infect*) adj3 (avert* or avoid* or prevent* or reduc* or stop*)).ti,ab,kw. (4845)

309 ((prefil* or pre-fill*) adj saline).ti,ab,kw. (40)

310 flush*.ab. /freq=3 (8037)

311 (PosiFlush* or SiteScrub* or SwabFlush*).mp. (28)

312 or/300-311 [CLABSI PREVENTION] (81217)

313 299 and 312 [CATHETERS/HUBS - CLABSI PREVENTION] (13355)

314 limit 313 to yr="2012-current" [DATE LIMIT APPLIED] (7191)

315 limit 314 to english [LANGUAGE LIMIT APPLIED] (6977)

316 315 use coch [CDSR RECORDS] (16)

317 111 or 236 or 284 or 316 [ALL DATABASES] (2801)

318 remove duplicates from 317 (**2563**) [**TOTAL UNIQUE RECORDS**]

319 318 use ppez [MEDLINE UNIQUE RECORDS] (1855)

320 318 use oemezd [EMBASE UNIQUE RECORDS] (200)

321 318 use cctr [CENTRAL UNIQUE RECORDS] (492)

322 318 use coch [CDSR UNIQUE RECORDS] (16)

**CLABSI Prevention Search Results**:

| **Database** | **Original** | **After Deduplication** |
| --- | --- | --- |
| MEDLINE | 1,855 | 1,853 |
| Embase | 200 | 199 |
| CENTRAL | 492 | 423 |
| CDSR | 16 | 6 |
| Total | 2,563 | 2,481 |
|  | |  |

Relevant CLABSI prevention studies identified are included within the present narrative review.
